# Clinical tools in the diagnosis of disorders of sex development: a switch from the hormonal to the genetics laboratory?

**DOI:** 10.1515/almed-2021-0072

**Published:** 2021-11-03

**Authors:** Rodolfo A. Rey

**Affiliations:** Centro de Investigaciones Endocrinológicas “Dr. César Bergadá” (CEDIE), CONICET – FEI – División de Endocrinología, Hospital de Niños Ricardo Gutiérrez, Buenos Aires, Argentina; Unidad de Medicina Traslacional, Hospital de Niños Ricardo Gutiérrez, Buenos Aires, Argentina; Universidad de Buenos Aires, Facultad de Medicina, Departamento de Histología, Embriología, Biología Celular y Genética, Buenos Aires, Argentina

**Keywords:** anti-Müllerian hormone, FSH, next-generation sequencing, oestradiol, testosterone

Traditionally, the vast majority of societies around the world have had a binary approach to sex. “Girl or boy?” has typically been the first question about a newborn. This vision deeply rooted in the society has undoubtedly shaped the medical approach throughout times in face of individuals in whom the genetic sex, the sex of the gonads and/or the appearance of the genitalia showed inconsistencies. Yet, it was only in 2005 that experts in the various fields related to these conditions, including paediatricians, endocrinologists, surgeons, geneticists, psychologists and patients’ organisations first met to critically appraise the management of intersex disorders from a broad perspective and reach a consensus on future directions. The “Chicago consensus” set the bases for the use of a new nomenclature by coining the term “Disorders of Sex Development (DSD)” and, more importantly, dealt with critical issues such as the investigation and management of patients with DSD by a multidisciplinary team, the recommendation on the assignment of a social gender to the newborn considering cultural aspects of the family and the need for prospective studies addressing long-term outcomes [1]. Despite the relatively short time elapsed since then, major scientific and societal changes have occurred, which lead to permanent revisions on the subject [2], [3], [4].

One particular issue that has revolutionised the approach of diagnosis of congenital conditions is the advent of high throughput technologies, such as next-generation sequencing (NGS). Until the first decade of the present century, the candidate-gene approach prevailed, based on the anatomic and hormonal features of the patient with DSD [5]. The molecular diagnosis yield was relatively poor, except for extremely typical conditions such as complete androgen insensitivity syndrome (CAIS), the persistent Müllerian duct syndrome (PMDS) or congenital adrenal hyperplasia (CAH) (Table 1), where pathogenic variants (“mutations”) in the genes respectively encoding the androgen receptor, anti-Müllerian hormone (AMH) or its receptor or the enzyme 21-hydro-xylase were found in the majority of the cases. Conversely, the diagnostic efficiency was low in patients with gonadal dysgenesis, partial androgen insensitivity, ovotesticular DSD or non-CAH 46,XX DSD. The use of gene panels in the clinical setting or of the whole exome or whole genome sequencing in the research setting has dramatically increased the attainment of an aetiologic diagnosis in patients with DSD [6].

**Table 1:** Genetic aetiologies of Disorders of Sex Development (DSD).

46,XY DSD	Sex chromosome DSD (45,X/46,XY – 46,XX/46,XY – etc.)	46,XX DSD
Gonadal dysgenesis	Gonadal dysgenesis Ovotesticular DSD	Testicular DSD Ovotesticular DSD
Defects of androgen synthesis (Leydig cell hypoplasia, Steroidogenic defects)		Excess of androgen synthesis (CAH, Aromatase deficiency)
Disorders of androgen action (Partial or complete AIS)		
Disorders of AMH synthesis or action (PMDS)		

Does this mean the end of the clinical characterisation of patients with ambiguous genitalia or discordance between the aspect of the external genitalia and the karyotype? The availability of NGS is still limited. But even when it will become widespread – following its constant cost reduction –, the interpretation of the big data yielded by exome or genome sequencing will rely on the validation of the underlying pathophysiology. Indeed, one of the major challenges of this technology is its capacity to determine the pathogenicity of the thousands of gene variants detected in one read, and adherence to consensus guidelines produced by professional associations, like the American College of Medical Genetics and Genomics and the Association for Molecular Pathology is warranted [7]. These guidelines stress the importance of both the preanalytical and the postanalytical procedures. In fact, to decide performing NGS a clinical characterisation of the patient is essential, and the deepest the phenotyping, the more efficient the diagnostic yield. Similarly, the finding of variants of unknown significance in unexpected genes can be more easily interpreted when a precise characterisation of the patient has been made, by using anatomical descriptions (clinical, imaging) and hormonal assessments, including sex steroids, AMH and gonadotrophins [8], [9], [10]. Achieving a precise genetic diagnosis leads to a better, personalised medical approach in many cases. However, faced with the impossibility of performing sophisticated genetic analyses, a clinical and endocrine characterisation may be very useful for the first steps in the management of a patient with DSD [8, 9]. Indeed, patients need to be considered case by case, according to their karyotype, age at presentation and appearance of the internal and external genitalia, as well as other non-reproductive features [10]. Furthermore, the contribution made by the progressive introduction of mass spectrometry for the accurate measurement of steroids in serum or urine samples shows the improvement in diagnosis in neonates [11] or in the therapeutic management later in life [12], [13], [14].

In conclusion, deep phenotyping by means of anatomic and endocrine characterisation remains essential for the initial diagnosis of DSD with either a 46,XX, 46,XY or other abnormal karyotypes. Enlarged panels of sex steroid measurement by mass spectrometry and massive parallel sequencing of potentially involved genes have come to improve the efficiency in aetiological diagnosis leading to an enhanced personalised medicine approach in DSD.

## References

[1] Lee PA, Houk CP, Ahmed SF, Hughes IA (2006). In collaboration with the participants in the International Consensus Conference on Intersex organized by the Lawson Wilkins Pediatric Endocrine Society and the European Society for Paediatric Endocrinology. Consensus Statement on Management of Intersex Disorders. Pediatrics.

[2] Lee PA, Nordenstrom A, Houk CP, Ahmed SF, Auchus R, Baratz A (2016). Global disorders of sex development update since 2006: perceptions, approach and care. Horm Res Paediatr.

[3] Cools M, Nordenström A, Robeva R, Hall J, Westerveld P, Flück C (2018). Caring for individuals with a difference of sex development (DSD): a consensus statement. Nat Rev Endocrinol.

[4] Ahmed SF, Achermann JC, Alderson J, Crouch NS, Elford S, Hughes IA (2021). Society for Endocrinology UK guidance on the initial evaluation of a suspected difference or Disorder of Sex Development (DSD) (revised 2021). Clin Endocrinol (Oxf).

[5] Rey RA, Grinspon RP (2011). Normal male sexual differentiation and aetiology of disorders of sex development. Best Pract Res Clin Endocrinol Metab.

[6] Delot EC, Vilain E (2021). Towards improved genetic diagnosis of human differences of sex development. Nat Rev Genet.

[7] Richards S, Aziz N, Bale S, Bick D, Das S, Gastier-Foster J (2015). Standards and guidelines for the interpretation of sequence variants: a joint consensus recommendation of the American College of Medical Genetics and Genomics and the Association for Molecular Pathology. Genet Med.

[8] Granada ML, Audí L (2021). El laboratorio en el diagnóstico multidisciplinar del desarrollo sexual anómalo o diferente (DSD) – I–II. Adv Lab Med.

[9] Granada ML, Audí L (2021). El laboratorio en el diagnóstico multidisciplinar del desarrollo sexual anómalo o diferente (DSD) – III–IV. Adv Lab Med.

[10] Grinspon RP, Castro S, Rey RA (2021). Up to date clinical and biochemical workup of the child and the adolescent with a suspected DSD. Horm Res Paediatr.

[11] Oliveira LR, Longui CA, Guaragna-Filho G, Costa JL, Lanaro R, Silva DA (2020). Androgens by immunoassay and mass spectrometry in children with 46,XY disorder of sex development. Endocr Connect.

[12] Flück CE, Meyer-Boni M, Pandey AV, Kempna P, Miller WL, Schoenle EJ (2011). Why boys will be boys: two pathways of fetal testicular androgen biosynthesis are needed for male sexual differentiation. Am J Hum Genet.

[13] Miller WL (2017). Disorders in the initial steps of steroid hormone synthesis. J Steroid Biochem Mol Biol.

[14] Jha S, Turcu AF, Sinaii N, Brookner B, Auchus RJ, Merke DP (2021). 11-Oxygenated androgens useful in the setting of discrepant conventional biomarkers in 21-hydroxylase deficiency. J Endocr Soc.

